# PSMA PET Imaging for Prostate Cancer Recurrence After Minimally Invasive Focal Therapy: A Single-Center Retrospective Study

**DOI:** 10.2967/jnumed.125.271556

**Published:** 2026-11

**Authors:** Mahbod Jafarvand, Andrea Farolfi, Riccardo Mei, Loic Djaileb, Matthias Benz, Alex Chung, Wayne Brisbane, Leonard Marks, Tristan Grogan, Andrei Gafita, Lela Theus, Andrew T. Nguyen, Johannes Czernin, Jeremie Calais

**Affiliations:** 1 Ahmanson Translational Theranostics Division, Department of Nuclear Medicine and Theranostics, UCLA, Los Angeles, California;; 2 Nuclear Medicine, IRCCS Azienda Ospedaliero-Universitaria di Bologna, Bologna, Italy;; 3 Nuclear Medicine Department, LRB, Université. Grenoble Alpes, INSERM, CHU Grenoble Alpes, Grenoble, France;; 4 Department of Radiological Sciences, UCLA, Los Angeles, California;; 5 Department of Urology, David Geffen School of Medicine, UCLA, Los Angeles, California;; 6 Department of Medicine Statistics Core, David Geffen School of Medicine, UCLA, Los Angeles, California; and; 7 Division of Nuclear Medicine and Molecular Imaging, Russell H. Morgan Department of Radiology and Radiological Science, Johns Hopkins University School of Medicine, Baltimore, Maryland

**Keywords:** PSMA, PET/CT, focal therapy, biochemical recurrence, mpMRI

## Abstract

Prostate-specific membrane antigen (PSMA)–targeted PET/CT (PSMA PET/CT) has become the first-line imaging modality used for staging and restaging prostate cancer. Its utility after minimally invasive focal therapy (high-intensity focused ultrasound, laser ablation, cryoablation) remains unclear. This study evaluated PSMA PET/CT for detecting and localizing recurrence after focal therapy. **Methods:** This retrospective single-center study included patients with prior focal therapy for prostate cancer who underwent ^68^Ga-PSMA-11 PET/CT for suspected recurrence between 2016 and 2023 without intervening treatment. Three masked readers analyzed whole-body PET/CT images using a per-region (prostate, pelvic nodes, distant metastases) majority rule for per-patient positivity. Interreader agreement was determined using Fleiss κ. In a subcohort of 39 patients with both MRI and biopsy results available within 3 mo of PSMA PET/CT, per-patient and per-segment (12 prostate segments) accuracy was assessed. One masked reader evaluated PET/CT at 60 min (whole-body) and 90 min (pelvic) postinjection, and one prostate MRI expert independently read the MRI scans. Biopsy pathology-confirmed clinically significant prostate cancer, defined as International Society of Urological Pathology grade 2 or greater, served as the reference standard. **Results:** In total, 112 patients were included. PSMA PET/CT showed 82% positivity for intraprostatic recurrence, 15% for pelvic nodes, and 9% for distant metastases. Interreader agreement was moderate (κ = 0.4; 95% CI, 0.32–0.54). In the subcohort, per-patient sensitivity was 85% for PET vs. 82% for MRI. All cases with an SUV_max_ of 10 or greater had an ISUP grade group of 3 or greater. **Conclusion:** PSMA PET/CT effectively localizes prostate cancer recurrence after focal therapy, outperforming MRI on sensitivity.

## Visual Abstract

**Figure:**
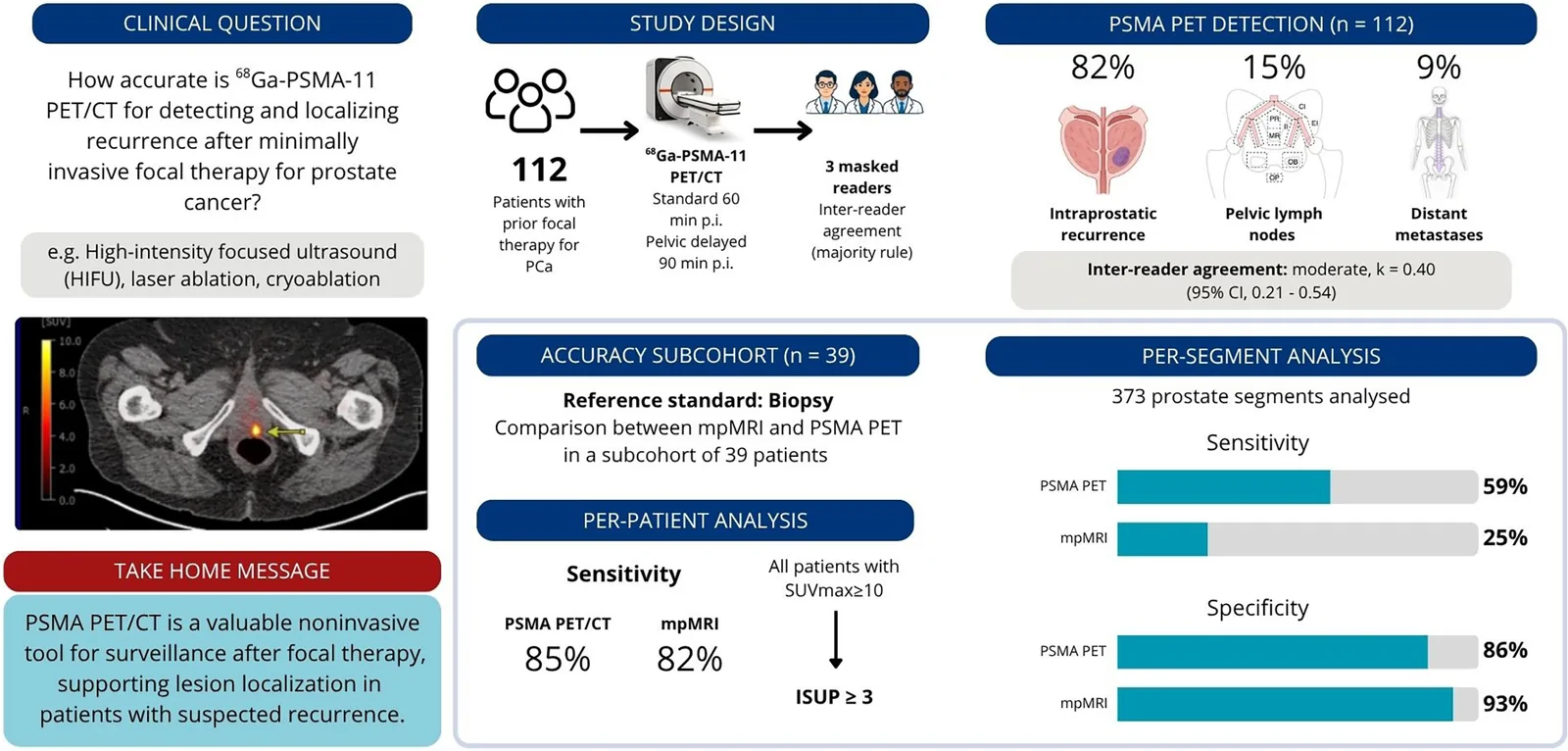

Prostate-specific membrane antigen (PSMA)–targeted PET/CT (PSMA PET/CT) has become the imaging modality of choice for staging and restaging prostate cancer (1). However, its role in localization of recurrence after minimally invasive focal therapy has not been defined yet. Focal therapy is an emerging alternative treatment modality for low- and intermediate-risk prostate cancers that aims to reduce the morbidity seen with radical therapy while maintaining cancer control. There are different methods of focal therapy, such as high-intensity focused ultrasound (HIFU), irreversible electroporation, photodynamic therapy, cryoablation, radiofrequency ablation, laser ablation, and focal brachytherapy (2). The rates of high-grade complications, such as rare incontinence and mild or transient reductions in erectile function, are low compared with those associated with radical treatment (3). Currently, no accurate method exists to evaluate the response to focal therapy. Patients can be followed by conventional methods, such as biopsies, prostate-specific antigen (PSA) and multiparametric MRI (mpMRI). Biopsy is the most accurate method for diagnosing residual disease or recurrence. However, it is an invasive procedure with associated risks and morbidity (4,5). The noninvasive methods (e.g., PSA monitoring, mpMRI) are not accurate enough. mpMRI interpretation is a major challenge because of signal alterations after focal therapy. A prior study of MRI after focal therapy described focal hemorrhages that limited mpMRI interpretation (6). The role of PSMA PET in patients who underwent focal therapy is still unknown. Few studies have evaluated the utility of PSMA PET/CT after focal therapy (7,8). In this study, we aimed to investigate the diagnostic efficacy of PSMA PET/CT to detect and localize recurrence or guide biopsy after focal therapy.

## MATERIALS AND METHODS

### Study Design and Patients

This single-center retrospective study was conducted at the University of California Los Angeles. Eligible patients had undergone minimally invasive focal therapy (HIFU, cryoablation, electroporation, or laser ablation), followed by PSMA PET/CT performed for suspected recurrence on the basis of PSA levels, with no intervening therapy between focal therapy and PSMA PET. Clinical data were extracted from electronic medical records by 2 local investigators. We assessed the per-patient and per-region positivity rates of the scans. In patients with available MRI and biopsy results performed within 3 mo of PSMA PET, we assessed the diagnostic efficacy performance parameters with histopathology as the reference standard on a per-patient and per-segment basis.

### PSMA PET Image Acquisition

^68^Ga-PSMA-11 (Glu-NH-CO-NH-Lys-(Ahx)-[^68^Ga (HBED-CC)]) was used as the PSMA tracer (9). The median injected activity was 5 mCi (range, 2.0–9.39 mCi) (125 MBq; range, 74–347.43 MBq). The median tracer uptake time was 62 min (range, 21–140 min).

We acquired images using a 64-detector PET/CT scanner (2007 Biograph 64 TruePoint or 2010 Biograph mCT 64; Siemens Healthineers). A diagnostic CT scan (200–240 mAs, 120 kV) was performed after the administration of oral contrast medium (600 mL of barium sulfate, 2.1% [Readi-Cat 2; Bracco]) and intravenous contrast medium (115 mL of iohexol [Omnipaque; GE HealthCare], 350 mg iodine/mL, injection speed of 2 mL/s, portal venous phase plus 80 s after injection) unless contraindicated. PET image acquisition included a whole-body scan (pelvis to vertex, 2–4 min/bed position, depending on the patient weight (10), and a dedicated pelvic scan after voiding 90 min postinjection (same acquisition time/bed position time as used for the whole-body scan). All PET images were reconstructed using attenuation, dead-time, random-event, and scatter corrections. PET images were reconstructed with an iterative algorithm (ordered-subset expectation maximization) in an axial 168 × 168 matrix on the Biograph 64 TruePoint (2-dimensional, 2 iterations, 8 subsets, gaussian filter 5.0) and in a 200 × 200 matrix on the Biograph mCT 64 (3-dimensional, 2 iterations, 24 subsets, gaussian filter 5.0).

### MR Image Acquisition

MRI was performed on a 3-T system (Magnetom Vida, Skyra, TrioTim, Prima Fit, or Verio; Siemens Healthineers). For signal reception, an external pelvic phased-array receiver coil was used. Conventional multiplanar T2-weighted turbo spin-echo imaging, axial unenhanced T1-weighted imaging, diffusion weight imaging, and axial 3-dimensional fast-field echo dynamic contrast-enhanced imaging were performed. Small-field-of-view 3-dimensional turbo spine-echo T2 weighted axial sequences were obtained using spatial and chemical-shift encoded excitation (Siemens Healthineers), as described previously (11,12).

### Image Analysis

Three independent masked readers analyzed the whole-body PET/CT images on a per-region basis (T, N, M1a, M1b, M1c) in accordance with the PROMISE V2 criteria (1). Because there is no strict guideline for determining positivity rate after focal therapy, interpretation was based on readers’ experience. A centralized majority rule was applied to generate the final positivity rate.

In patients with MRI and biopsy performed within 3 mo of PSMA PET, the scans were evaluated on a per-patient and per-segment basis. Twelve prostatic segments were defined (right anterior apex, midgland, and base; left anterior apex, midgland, and base; right posterior apex, midgland, and base; and left posterior apex, midgland, and base). One independent PET reader performed the analysis on both the standard whole-body images (acquired after 60 min) and the delayed pelvic images (acquired after 90 min). The SUV_max_ of the prostate findings was measured on both standard and delayed PET images.

One prostate MRI radiologist masked to PSMA PET and pathology findings interpreted the mpMRI results using Prostate Imaging Reporting and Data System version 2.1.

### Histopathology of Prostate Biopsies

Systematic and targeted transrectal ultrasound and MR/ultrasound fusion biopsies were performed by experienced urologists per the standard of care. Clinical pathology reports were retrospectively reviewed to assess for every segment in which recurrence was suspected. The biopsy cores were grouped into the 12 segments described above for comparison with the PSMA PET/CT results. Clinically significant prostate cancer was considered International Society of Urological Pathology grade 2 or greater.

### Statistical Analysis

Patient characteristics were summarized using median (interquartile range) for continuous variables and frequency (percentage) for categoric variables, reported for both the overall cohort (*n* = 112) and the diagnostic accuracy subcohort (*n* = 39). The primary outcome was PSMA PET/CT positivity, assessed on a per-patient and per-segment basis using a centralized majority rule across 3 independent readers. Interreader agreement for prostate positivity was evaluated using Fleiss κ. In the diagnostic subcohort with matched biopsy and MRI, we calculated sensitivity, specificity, positive predictive value (PPV), and negative predictive value (NPV) for PSMA PET/CT (at 60 and 90 min) and mpMRI, using biopsy histopathology as the reference. Sensitivity and specificity were compared between PET and MRI using McNemar test. Because PPV and NPV are prevalence-dependent, differences between modalities were evaluated using nonparametric bootstrapping with 1000 resamples to estimate 95% CIs and *P* values. All analyses were performed in R version 4.3.2 (R Foundation for Statistical Computing), and *P* values of less than 0.05 were considered statistically significant.

## RESULTS

### Patient Population

In total, 3341 patients were retrospectively screened, including 450 patients who underwent focal therapy between 1998 and 2022 and 3243 patients who underwent PSMA PET/CT between 2016 and 2023. Of these, 112 patients met the inclusion criteria (Fig. 1). The median age was 70 y (interquartile range [IQR], 64–76 y). Among the 112 patients, 58 (52%) underwent HIFU, 43 (38%) underwent cryoablation, and 11 (10%) underwent either laser ablation or electroporation. The median PSA at the time of PSMA PET/CT was 5.2 ng/mL (IQR, 2.8–9.0 ng/mL). The median interval between focal therapy and PSMA PET/CT was 27 mo (IQR, 15–45 mo). The institutional review board approved this retrospective study, and the requirement to obtain informed consent was waived (IRB 22-000867). Detailed patient characteristics are summarized in Table 1.

**FIGURE 1.**
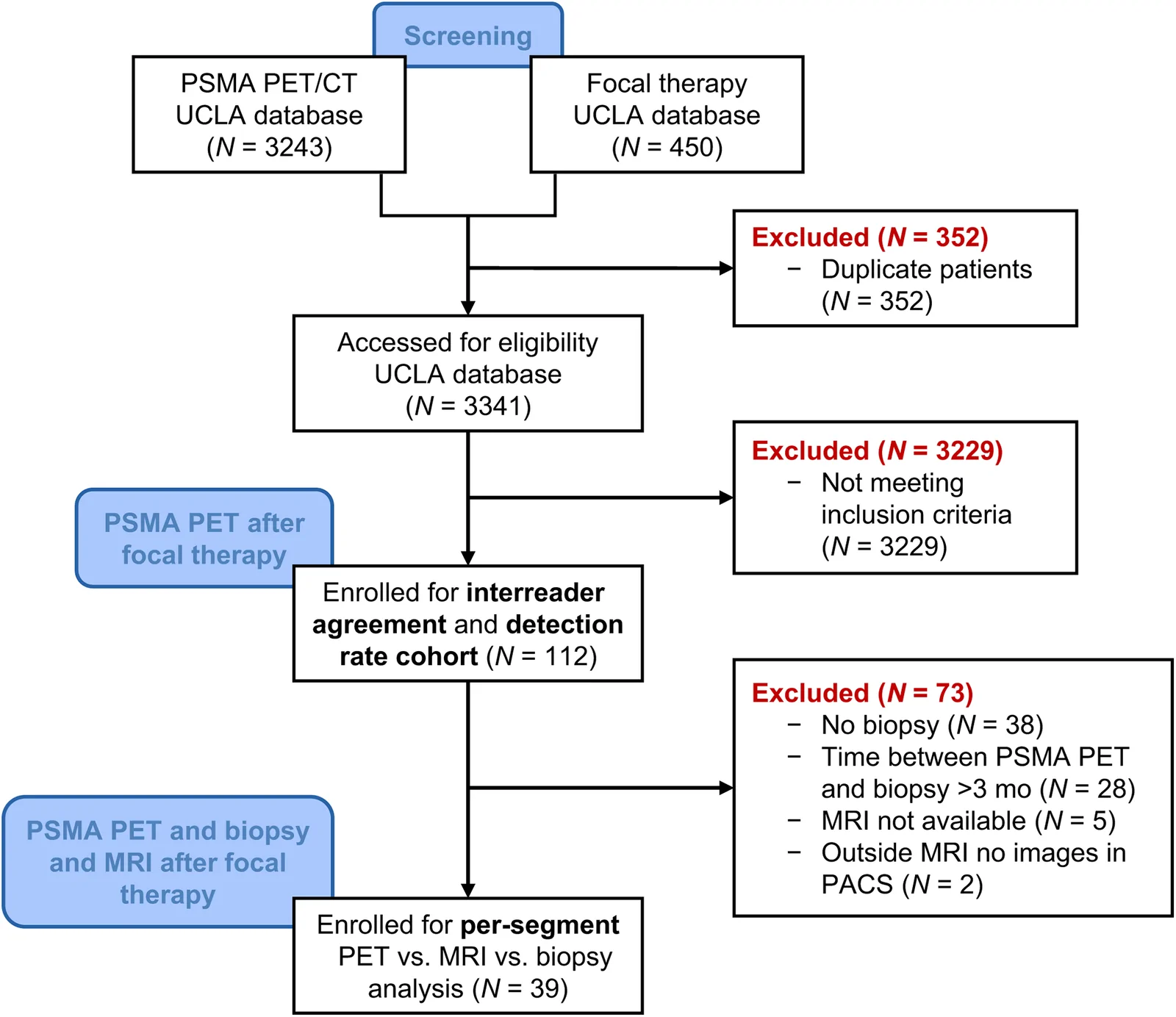
Consolidated Standards of Reporting Trials (CONSORT) diagram for patient selection.

**TABLE 1.** Patient Characteristics of Main Cohort (Detection Rate Analysis) and MRI/Biopsy Cohort (Diagnostic Accuracy Analysis)

Characteristic	Main cohort	MRI–biopsy cohort
*n*	112	39
Age (y)	70 (64–76)	71 (64.5–76)
Initial PSA (ng/mL)	6.8 (5.2–10)	7.5 (5.4–10.6)
Initial ISUP (Gleason score)		
1 (3 + 3)	17 (15)	5 (13)
2 (3 + 4)	40 (36)	19 (49)
3 (4 + 3)	26 (23)	10 (25)
4 (3 + 5) (4 + 4)	15 (13)	3 (8)
5 (4 + 5) (5 + 4)	3 (3)	2 (5)
NA	11 (10)	
Initial NCCN risk score		
Very low risk	14 (13)	5 (13)
Low risk	0	0
Intermediate risk	79 (70)	31 (79)
High risk	8 (7)	3 (8)
Very high risk	0	0
NA	11 (10)	
Type of focal therapy		
HIFU	58 (52)	20 (51)
Cryoablation	43 (38)	15 (38)
Others (electroporation, laser)	11 (10)	4 (10)
PSA at PET scan (ng/mL)	5.2 (2.8–9)	5.5 (3.6–9)
Time between focal therapy and PSMA PET (mo)	27 (15–44.8)	25 (14–39)
Time between focal therapy and biopsy (mo)		25 (10–39)
Time between focal therapy and MRI (mo)		19 (14–35)
Time between PET and biopsy (d)		35 (20–52)
Time between PET and MRI (d)		57 (33–131)
ISUP at biopsy		
Negative		3 (8)
1 (Gleason score ≤6)		2 (5)
2 (Gleason score 3 + 4)		8 (21)
3 (Gleason score 4 + 3)		13 (33)
4 (Gleason score 8)		4 (10)
5 (Gleason score 9 or 10)		9 (23)

ISUP = International Society of Urological Pathology; NCCN = National Comprehensive Cancer Network.

Qualitative data expressed as number, followed by percentage in parentheses; continuous data expressed as median, followed by interquartile range in parentheses.

### Scan Positivity Rate and Interreader Agreement

On the basis of majority reads, PSMA PET/CT was positive in 92 patients (82%) on a per-patient basis (Table 2). Of the 112 patients, 92 (82%) demonstrated intraprostatic uptake, 3 (3%) had suspected extracapsular extension (T3a), and 27 (24%) showed suspected seminal vesicle invasion (T3b). Pelvic lymph node involvement was identified in 17 patients (15%), and distant metastases were suspected in 10 patients (9%).

**TABLE 2.** Per-Patient PSMA PET Positivity Rate Categorized by PROMISE Subtype and SUV_max_ (*n* = 112)

		SUV_max_	
PROMISE Subtype	Positivity Rate	60 min	90 min
Positive PSMA PET	92 (82)		
Prostate (T)	92 (82)	7.5 (5.2–12.8)	8.4 (5.8–15.4)
T2	83 (74)		
Extracapsular (T3a)	3 (3)		
Seminal vesicles (T3b)	27 (24)		
Pelvic lymph nodes (N)	17 (15)	9.1 (4.0–13.8)	
Distant metastases (M)	10 (9)	8.2 (5.2–26.7)	
Distant lymph nodes (M1a)	3 (3)		
Bone (M1b)	8 (7)		
Visceral (M1c) (lung)	1 (1)		

Values based on majority rule.

Qualitative data expressed as number, followed by percentage in parentheses; continuous data expressed as median, followed by interquartile range in parentheses.

Perfect agreement across all 3 readers was observed for 81 cases (72%). Reader disagreement occurred in 31 cases (28%), with a Fleiss κ of 0.4 (95% CI, 0.32–0.54), indicating moderate interreader agreement (Supplemental Table 1, available at http://jnm.snmjournals.org). Notably, 28 of these 31 cases involved patients with 0 or 1 prostate lesion and PRIMARY scores of no more than 4. Detailed individual reader results are provided in Supplemental Table 2.

### Diagnostic Accuracy with Histopathology Reference Standard

Diagnostic accuracy analysis was conducted for a subset of 39 patients who underwent both mpMRI and prostate biopsy within 3 mo of PSMA PET/CT. In this subgroup, 20 patients (51%) were treated with HIFU, 15 (38%) with cryoablation, and 4 (10%) with laser or electroporation. The median PSA at the time of PSMA PET/CT was 5.5 ng/mL (IQR, 3.6–9.0 ng/mL). The median interval between focal therapy and biopsy was 25 mo (IQR, 14–39 mo), with a median interval of 35 d (IQR, 20–52 d) between PSMA PET/CT and biopsy and 57 d (IQR, 33–131 d) between MRI and biopsy (Table 1).

Among these patients, 34 (87%) had clinically significant prostate cancer confirmed on biopsy. A total of 373 prostate segments (of a possible 468) had matched biopsy data and were included in the segment-level analysis. Further, 151 segments (41%) were positive for prostate cancer (ISUP grade group ≥ 2) and 222 (59%) were negative (ISUP grade group < 2), with the following Gleason score distribution: 6 or lower (*n* = 16, 4%), 3 + 4 (*n* = 31, 8%), 4 + 3 (*n* = 45, 12%), 8 (*n* = 21, 6%), and 9 or 10 (*n* = 54, 15%). Detailed per-patient read and pathology results provided in (Supplemental Table 3).

#### Per-Patient Analysis

By majority reads, PSMA PET/CT correctly identified 29 true positives, 2 true negatives, 3 false positives, and 5 false negatives (Supplemental Table 4). On a per-patient basis, the sensitivity, specificity, PPV, and NPV were 85%, 40%, 91%, and 29%, respectively (Table 3).

**TABLE 3.** Per-Patient Diagnostic Performance Values

	Value (95% CI)		
Variable	PSMA PET	MRI	*P*
Sensitivity	85% (69%–95%)	82% (65%–93%)	1.00
Specificity	40% (5%–85%)	20% (1%–72%)	1.00
PPV	91% (82%–95%)	88% (81%–92%)	0.326
NPV	29% (9%–61%)	14% (2%–53%)	0.484

Values based on 3 PET readers and majority rule and independent MRI reader using biopsy as reference standard.

MRI identified 28 true positives, 1 true negative, 4 false positives, and 6 false negatives (Supplemental Table 5). On a per-patient basis, the sensitivity, specificity, PPV, and NPV were 82%, 20%, 88%, and 14%, respectively (Table 3).

#### Per-Segment Analysis

Segment-level analysis was performed by one PET reader.

PSMA PET at 60 min yielded 89 true positives, 181 true negatives, 41 false positives, and 62 false negatives (Supplemental Table 6), corresponding to a sensitivity, specificity, PPV, and NPV of 59%, 82%, 68%, and 74%, respectively. At 90 min, PSMA PET demonstrated 89 true positives, 191 true negatives, 31 false positives, and 62 false negatives (Supplemental Table 7), with diagnostic performance of 59%, 86%, 74%, and 75%, respectively.

By comparison, MRI identified 38 true positives, 206 true negatives, 16 false positives, and 113 false negatives (Supplemental Table 8), resulting in a sensitivity of 25%, specificity of 93%, PPV of 70%, and NPV of 65% (Table 4).

**TABLE 4.** Segment-Based Diagnostic Performance Values

	Value (95% CI)			*P*	
Per-segment analysis	Standard PET (60 min)	Standard + delayed PET (60 + 90 min)	MRI	Standard PET vs. MRI	Delayed PET vs. MRI
Sensitivity	59% (51%–67%)	59% (51%–67%)	25% (18%–33%)	<0.001	<0.001
Specificity	82% (76%–86%)	86% (81%–90%)	93% (89%–96%)	<0.001	0.029
PPV	68% (62%–75%)	74% (67%–80%)	70% (58%–80%)	0.810	0.470
NPV	74% (70%–78%)	75% (71%–79%)	65% (62%–67%)	<0.001	<0.001

Values based on 1 PET reader and an independent MRI reader using biopsy as reference standard.

Representative imaging examples are provided to illustrate true-positive (Fig. 2), false-positive (Fig. 3), and false-negative (Fig. 4) findings of PSMA PET/CT and MRI in patients undergoing evaluation after focal therapy.

**FIGURE 2.**
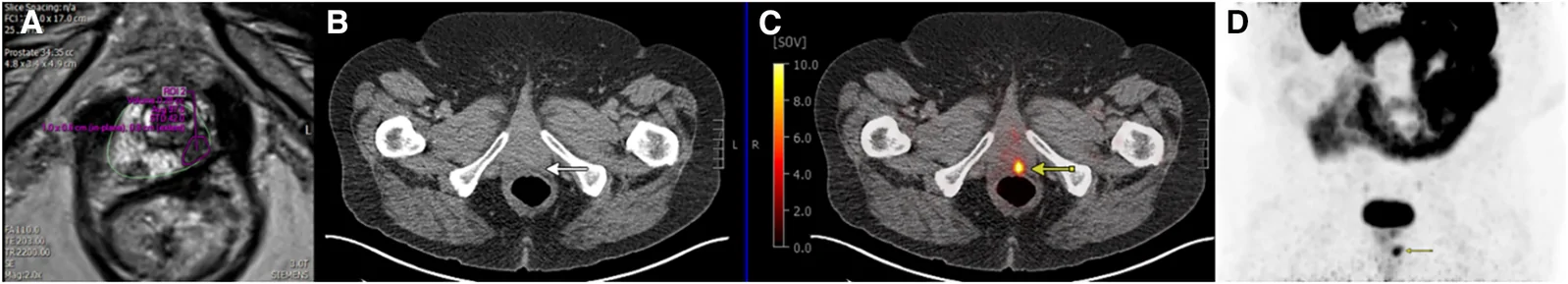
True-positive PSMA PET with positive MRI in 74-y-old male with prostate cancer (Gleason score of 4 + 5; left anterior and posterior apex positive cores). (A) Left posterior peripheral apical to midprostate hyposignal visible on transaxial MRI. (B) No definite CT correlate evident (white arrow). (C) Focal high PSMA expression detectable in left posterior mid to base with SUV_max_ of 12.4 on transaxial fused delayed pelvic PET/CT (yellow arrow). (D) Focal high PSMA expression found in left midgland to base of prostate on maximum-intensity-projection image (yellow arrow).

**FIGURE 3.**
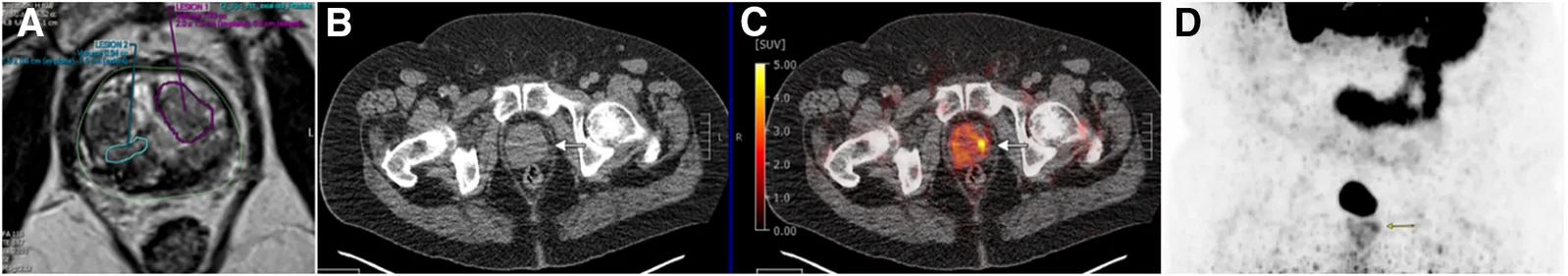
False-positive PSMA PET/CT and MRI in 67-y-old male with negative biopsy. (A) Bilateral midprostate hyposignal visible on transaxial MRI. (B) Tiny hypodense focus evident in left peripheral midgland on transaxial CT. (C) Tiny focal high PSMA expression seen in left peripheral midgland with SUV_max_ of 5.2 on transaxial fused delayed pelvic PET/CT (90 min). (D) Tiny focal high PSMA expression visible in left posterior peripheral mid gland on maximum-intensity-projection image.

**FIGURE 4.**
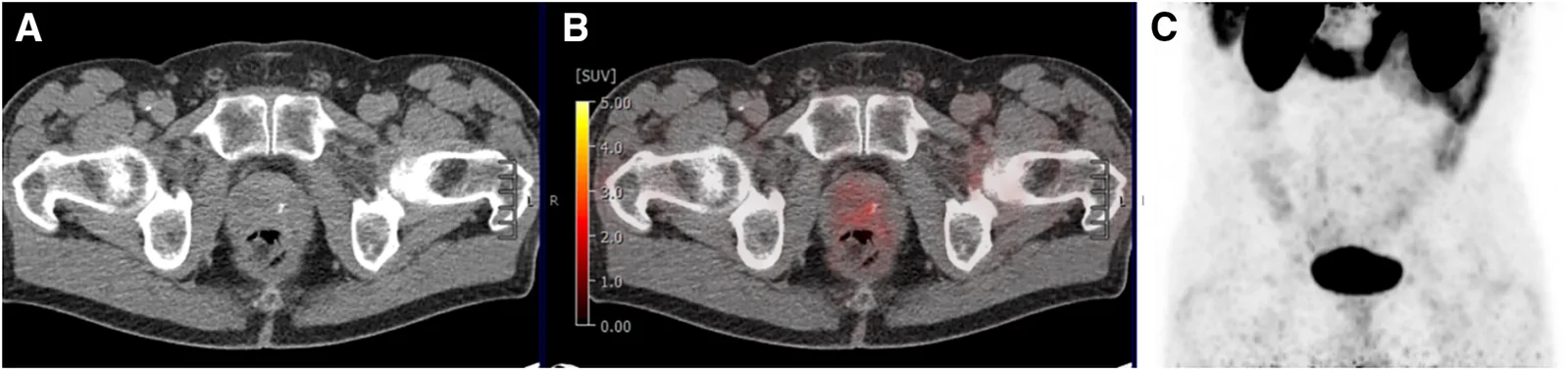
False-negative PSMA PET/CT and MRI in 60-y-old male with prostate cancer (Gleason score of 4 + 4; left apical anterior and posterior positive cores). (A) Right apical hypodensity visible on transaxial CT. (B) Heterogeneous low PSMA expression without focality in left prostate (90-min PSMA PET). (C) Heterogeneous low PSMA expression within prostate on maximum-intensity-projection image.

Statistical comparisons demonstrated that PSMA PET at 60 min had significantly higher sensitivity compared with MRI (59% vs. 25%, *P* < 0.001), whereas MRI showed higher specificity (93% vs. 82%, *P* < 0.001). PPV was comparable between modalities (68% vs. 70%, *P* = 0.81), whereas NPV favored PSMA PET (74% vs. 65%, *P* < 0.001). Similar findings were observed when comparing PSMA PET at 90 min with MRI—sensitivity was superior for PSMA PET (59% vs. 25%, *P* < 0.001), specificity was slightly higher for MRI (93% vs. 86%, *P* = 0.029), PPV was similar (74% vs. 70%, *P* = 0.47), and NPV was greater for PSMA PET (75% vs. 65%, *P* < 0.001). Comparisons between PSMA PET at 60 and 90 min showed no significant differences in specificity (82% vs. 86%), PPV (68% vs. 74%), or NPV (74% vs. 75%).

### PSMA PET Uptake

The median SUV_max_ of prostate lesions was 7.5 (IQR, 5.2–12.8) on standard acquisition (*n* = 130) and 8.4 (IQR, 5.8–15.4) on delayed pelvic acquisition (*n* = 120). Notably, all patients with an SUV_max_ of 10 or greater had a ISUP grade group of 3 or greater on histopathology.

## DISCUSSION

In this study, we evaluated the diagnostic performance of PSMA PET/CT in detecting recurrent prostate cancer after focal therapy. PSMA PET demonstrated higher sensitivity and NPV compared with mpMRI, whereas MRI maintained superior specificity. These findings highlight complementary diagnostic profiles—PSMA PET may be better suited for identifying or excluding residual disease, whereas MRI may be more reliable for confirming true negatives in highly specific settings. These results are consistent with prior reports. Burger et al. demonstrated that PSMA PET/MRI could localize recurrent prostate cancer after HIFU, particularly in cases where mpMRI returned a false-negative result (7).

Despite the improved sensitivity, the interreader agreement in our study was only moderate (Fleiss κ = 0.4), which contrasts with higher concordance rates reported in earlier studies, such as those by Fendler et al. (13) and Bundschuh et al. (14). A possible explanation for the variability in reader interpretation is the complex posttreatment anatomy, as well as the presence of physiologic urinary excretion.

On a segment-level analysis, PSMA PET/CT showed high specificity, comparable with that of MRI. This supports its potential utility for lesion localization and targeted biopsy guidance. Statistical comparisons between early (60 min) and delayed (90 min) PSMA PET imaging showed no significant differences in specificity (82% vs. 86%) or PPV (68% vs. 74%), likely related in part to the limited sample size of this exploratory subcohort. Nevertheless, from a practical image interpretation standpoint, the PET expert readers felt that delayed pelvic bed imaging may provide added clinical value, particularly in cases complicated by urinary tracer activity, posttreatment inflammation, tissue remodeling, or treatment-related cavities. Delayed pelvic acquisition may help distinguish persistent focal uptake from urinary activity or nonspecific posttreatment changes, improve delineation of true intraprostatic lesions, and reduce equivocal findings in the prostate bed, consistent with prior literature. In addition, combining delayed pelvic PET acquisition with contrast-enhanced CT including an excretory phase may further assist in differentiating urinary structures from recurrent disease in equivocal cases (15). Larger prospective studies are needed to confirm the clinical value of delayed pelvic imaging in this setting.

Interpretation of intraprostatic PSMA uptake after focal therapy remains challenging because urinary activity, inflammation, benign prostatic hyperplasia, tissue remodeling, and treatment-related cavities may cause nonspecific uptake and mimic local recurrence. In this study, image interpretation was performed visually in accordance with the PROMISE criteria, with particular attention given to uptake focality, asymmetry, intensity, and concordance with CT findings. Delayed pelvic imaging and contrast-enhanced CT were often helpful in equivocal cases. Importantly, most reader disagreement cases (28 of 31) occurred in patients with 0 or 1 prostate lesion and PRIMARY scores of 4 or lower, highlighting that discordant interpretations were mainly related to low-volume or equivocal intraprostatic findings. Future studies should evaluate the utility of the PRIMARY scoring system (16) after focal therapy, as it incorporates both uptake pattern focality and SUV intensity. In addition, precise knowledge of the location and extent of prior focal therapy is important to better understand posttreatment tissue remodeling and treatment-related cavities that may affect image interpretation.

A recent study by Lopci et al. proposed the integration of PSMA PET imaging with MRI to overcome interpretation challenges associated with imaging after focal therapy, particularly the confounding effects of fibrosis and necrosis that hinder reliable detection of recurrence (17). To improve diagnostic confidence and reduce unnecessary biopsies, their group developed a novel PSMA PET–based scoring system specifically designed for assessment after focal therapy. Of note, an ongoing trial (NCT07185165) is exploring the role of PSMA PET systematically before and after focal therapy in patients with prostate cancer.

Our study had several limitations. First, this study was retrospective in nature and solely included patients referred to urology and nuclear medicine clinics for biochemical recurrence. This inherently introduced substantial selection bias, as patients were not undergoing systematic screening after focal therapy but rather targeted evaluation on the basis of concerning clinical, biochemical, or imaging findings. As a result, the biopsy subcohort was enriched for recurrent disease, with a high prevalence of clinically significant prostate cancer and a limited number of true-negative cases, thereby limiting the robustness and generalizability of specificity estimates. Additionally, only about one-third of our patients had contemporaneous MRI and biopsy results available, which limited the size of the per-patient and per-segment validation cohort. Another limitation was that mpMRI examinations were interpreted by a single reader, and 60- and 90-min pelvic PET acquisitions were evaluated by just one PET reader because of resource constraints. Therefore, the MRI–biopsy subcohort analysis represented a 1-to-1 reader comparison study without multireader assessment for MRI or delayed PET imaging. Future prospective studies with dedicated multireader designs are necessary to further validate these findings. False positives on PSMA PET remain a challenge, emphasizing the value of multimodal assessment and careful correlation with pathology. Interreader agreement for PSMA PET was moderate, underscoring the need for standardized interpretation frameworks and reader training. Furthermore, standardized criteria for defining biochemical recurrence after focal therapy are not yet established. In our study, recurrence was defined by PSA values rising above the posttreatment nadir.

## CONCLUSION

This study supports the clinical value of PSMA PET/CT in detecting recurrent prostate cancer after focal therapy, demonstrating high sensitivity and favorable diagnostic performances compared with MRI. These findings suggest that PSMA PET/CT may serve as a valuable tool for guiding posttreatment evaluation and biopsy planning in this patient population. Prospective studies with larger cohorts are warranted to validate these results and further define the optimal role of PSMA PET/CT after focal therapy.

## DISCLOSURE

The views expressed in this article are those of the authors and do not necessarily reflect the official policy or position of any affiliated institutions. No financial relationships, commercial affiliations, or personal associations influenced the work reported in this study. All relationships below for each author are outside the submitted work. Jeremie Calais reports grants from support to his institution from Lantheus, Novartis, and POINT Biopharma. He also reports consulting activities (advisory board member, speaker, blinded reader) for Advanced Accelerator Applications, Amgen, Astellas, Bayer, Blue Earth Diagnostics Inc., Curium Pharma, DS Pharma, Fibrogen, GE HealthCare, Isoray, IBA RadioPharma, Janssen Pharmaceuticals, Monrol, Lightpoint Medical, Lantheus, Novartis, Pfizer, POINT Biopharma, Progenics, Radiomedix, Sanofi, Siemens-Varian, SOFIE, and Telix Pharmaceuticals, outside the submitted work. Johannes Czernin is a scientific advisor and founder of SOFIE Biosciences and Infinity Topco; founder of Trethera Therapeutics; and scientific advisor for Aktis Oncology. Leonard Marks reports stock and other ownership interests with Avenda Health. No potential other conflict of interest relevant to this article was reported.

## Notes

KEY POINTS **QUESTION:** Can PSMA PET/CT accurately detect and localize recurrent prostate cancer after minimally invasive focal therapy? **PERTINENT FINDINGS:** In this single-center, retrospective cohort study of 112 patients evaluated after focal therapy, PSMA PET/CT showed suspected intraprostatic recurrence in 82% of cases and nodal or distant disease in 24%. In a biopsy-validated subcohort (*n* = 39), PSMA PET/CT demonstrated higher sensitivity than did mpMRI for detecting recurrent disease at both the patient and segment levels, although delayed pelvic imaging may help in specific cases. **IMPLICATIONS FOR PATIENT CARE:** PSMA PET/CT may provide a valuable noninvasive tool for surveillance after focal therapy, supporting lesion localization and targeted biopsy planning in patients with suspected recurrence.
